# Supplementary material for: Risk-Reducing Mastectomy and Reconstruction Following Prophylactic Breast Irradiation: Hope Sustained
Source: Cancers (Basel). 2021 May 30;13(11):2694. doi: 10.3390/cancers13112694 (PMC8198915; doi:10.3390/cancers13112694)

Figure S1. Flow Chart

Prophylactic Irradiation to the Contralateral Breast for BRCA Mutation Carriers with Early Mammary Cancer

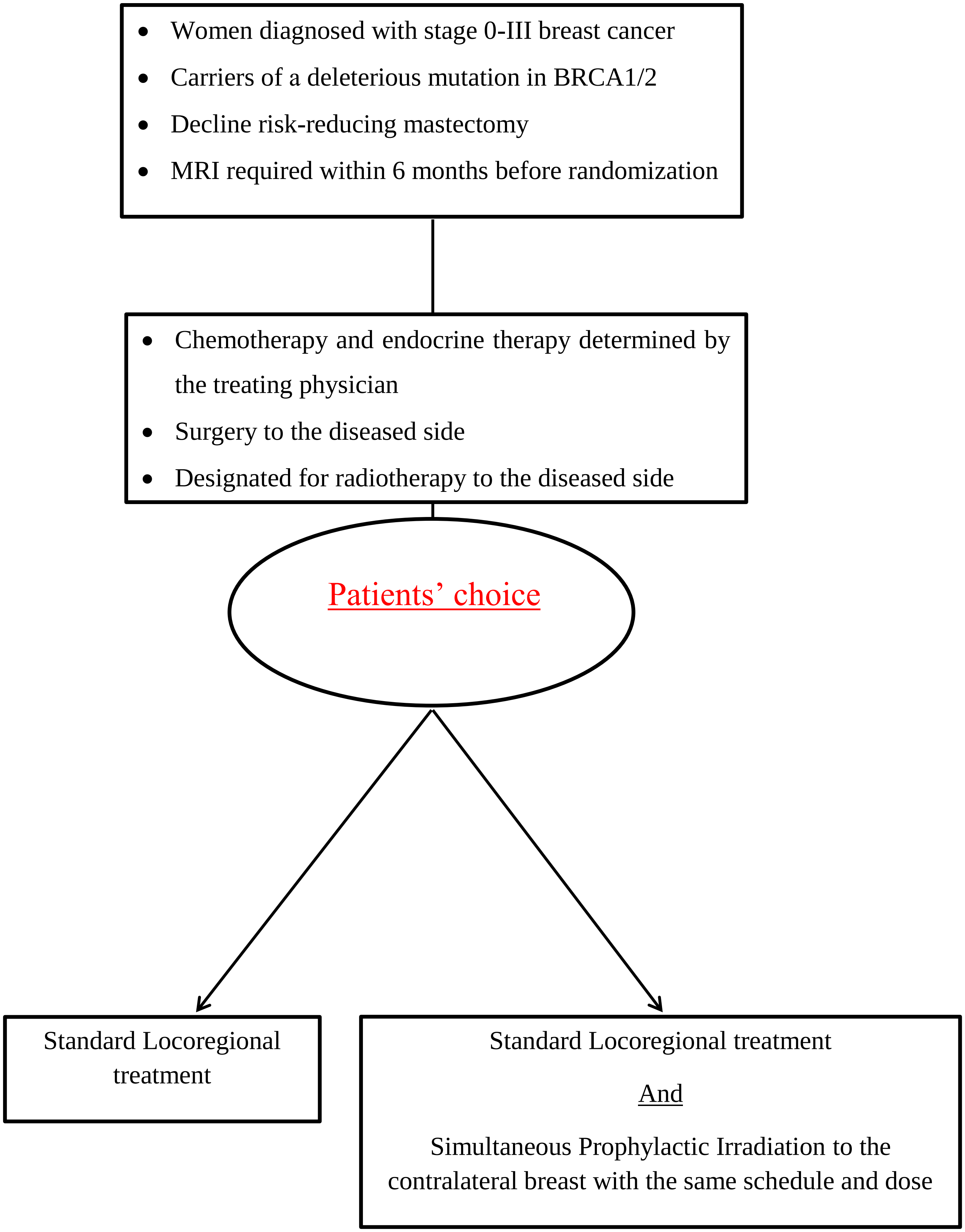

Supplement: Supplementary file 1 [file cancers-13-02694-s001.zip › cancers-1172820-supplementary.pdf]
